# Human mesenchymal stem cells enhance the systemic effects of radiotherapy

**DOI:** 10.18632/oncotarget.5216

**Published:** 2015-08-17

**Authors:** Virgínea de Araújo Farias, Francisco O'Valle, Borja Alonso Lerma, Carmen Ruiz de Almodóvar, Jesús J. López-Peñalver, Ana Nieto, Ana Santos, Beatriz Irene Fernández, Ana Guerra-Librero, María Carmen Ruiz-Ruiz, Damián Guirado, Thomas Schmidt, Francisco Javier Oliver, José Mariano Ruiz de Almodóvar

**Affiliations:** ^1^ Instituto Universitario de Investigación en Biopatología y Medicina Regenerativa, Centro de Investigación Biomédica, Universidad de Granada, Granada, Spain; ^2^ Instituto de Parasitología y Biomedicina “López Neyra”, Consejo Superior de Investigaciones Científicas, Granada, Spain; ^3^ Biochemistry Center, University of Heidelberg, Heidelberg, Germany; ^4^ Unidad de radiología experimental, Centro de Instrumentación Científica, Centro de Investigación Biomédica, Universidad de Granada, Granada, Spain; ^5^ Unidad de Experimentación Animal, Centro de Instrumentación Científica, Centro de Investigación Biomédica, Universidad de Granada, Granada, Spain; ^6^ Unidad de Microscopia, Centro de Instrumentación Científica, Centro de Investigación Biomédica, Universidad de Granada, Granada, Spain; ^7^ Departamento de Bioquímica y Biología Molecular III e Inmunología, IBIMER, Centro de Investigación Biomédica, Universidad de Granada, Granada, Spain; ^8^ Hospital Universitario San Cecilio, Granada, Spain; ^9^ Department of General, Visceral and Transplant Surgery, University of Heidelberg, Heidelberg, Germany

**Keywords:** radiotherapy, bystander effect, mesenchymal cells, cancer, radiosensitizers

## Abstract

The outcome of radiotherapy treatment might be further improved by a better understanding of individual variations in tumor radiosensitivity and normal tissue reactions, including the bystander effect. For many tumors, however, a definitive cure cannot be achieved, despite the availablity of more and more effective cancer treatments. Therefore, any improvement in the efficacy of radiotherapy will undoubtedly benefit a significant number of patients.

Many experimental studies measure a bystander component of tumor cell death after radiotherapy, which highlights the importance of confirming these observations in a preclinical situation. Mesenchymal stem cells (MSCs) have been investigated for use in the treatment of cancers as they are able to both preferentially home onto tumors and become incorporated into their stroma. This process increases after radiation therapy. In our study we show that *in vitro* MSCs, when activated with a low dose of radiation, are a source of anti-tumor cytokines that decrease the proliferative activity of tumor cells, producing a potent cytotoxic synergistic effect on tumor cells. *In vivo* administration of unirradiated mesenchymal cells together with radiation leads to an increased efficacy of radiotherapy, thus leading to an enhancement of short and long range bystander effects on primary-irradiated tumors and distant-non-irradiated tumors. Our experiments indicate an increased cell loss rate and the decrease in the tumor cell proliferation activity as the major mechanisms underlying the delayed tumor growth and are a strong indicator of the synergistic effect between RT and MSC when they are applied together for tumor treatment in this model.

## INTRODUCTION

Our knowledge of the mechanisms via which radiation induces cell death is based on data of cell survival and cell damage after radiation [1-3] and on the consequences that this damage generates at a cellular [4], tumoral and normal tissue level [5, 6]. In radiation oncology a growing need exists for the development of clinical-decision-support systems based on prediction models of treatment outcome [7]. The models proposed so far to explain the outcome of tumor growth and adverse effects of radiotherapy have led to the current knowledge that not only DNA damage, but also cell signaling processes in off-target cells (bystander and ascopal effects) [8-11] can be crucial to the effect of radiotherapy [12, 13]. New models should combine both predictive and prognostic data factors from clinical, imaging, molecular and other sources to achieve the highest accuracy to predict tumour response and follow-up event rates [13, 14]. Consequently, models which include the bystander effect would seem to be necessary [15]. Indeed, cell-cell communication between sub-lethally damaged cells after radiotherapy and surviving tumor cells leads to a reduction in the remaining and viable cancer cells [16]. Therefore, non-targeted radiation effects might be considered as the response of the tumor [17, 18] and normal tissues [6, 19] to the stress induced by radiation in the target volume [20].

Mesenchymal stem cells (MSC) [21] have been investigated for the treatment of cancers as they are able to home onto tumors and become incorporated into their stroma. Moreover, MSC homing is increased after radiation therapy [22]. MSCs can both suppress or promote tumor growth [23-25]. The existing information proposes that, as a response to injury, MSCs might have a role in regenerating tissues. This process occurs upon the activation of these MSCs, which become mobilized, activated and secrete factors that enable a cell therapy microenviroment [26, 27] and the molecules secreted by the activated MSCs (MSCs*) may affect a variety of immune cell lineages and establish a powerful therapeutic field [28, 29]. Taking into account both previous reports and our own experience we have designed this study to investigate the following hypothesis:

“The radiotherapy itself will not be systemic although radiotherapy may contribute to a systemic effect”.

To check this hypothesis, we have investigated cellular sensitivity to the bystander effect, using a set of cancer cell lines and mesenchymal cells derived from umbilical cord stroma, including the activation of MSCs with radiotherapy.

Our *in vitro* and *in vivo* findings show that: 1) TRAIL and DKK3 are molecules produced by mesenchymal cells that, as a consequence of the cell treatment with low-LET radiation at low doses, are secreted to the extracellular space where they can act as signaling molecules to produce tumor cell death and 2) the activation of MSCs with radiotherapy at low doses may be a useful tumor-suppressor strategy for the treatment of cancer based on the intercellular communication of these cells with neigboring tumor cells to reach distant localizations, both via physical contact and lymphatic and circulatory networks, and produce cell loss in off-target tumor cells.

## RESULTS

### Tumor cells exposed to MSC radiation conditioned medium (RCM) show a reduction in survival

**Figure 1:**
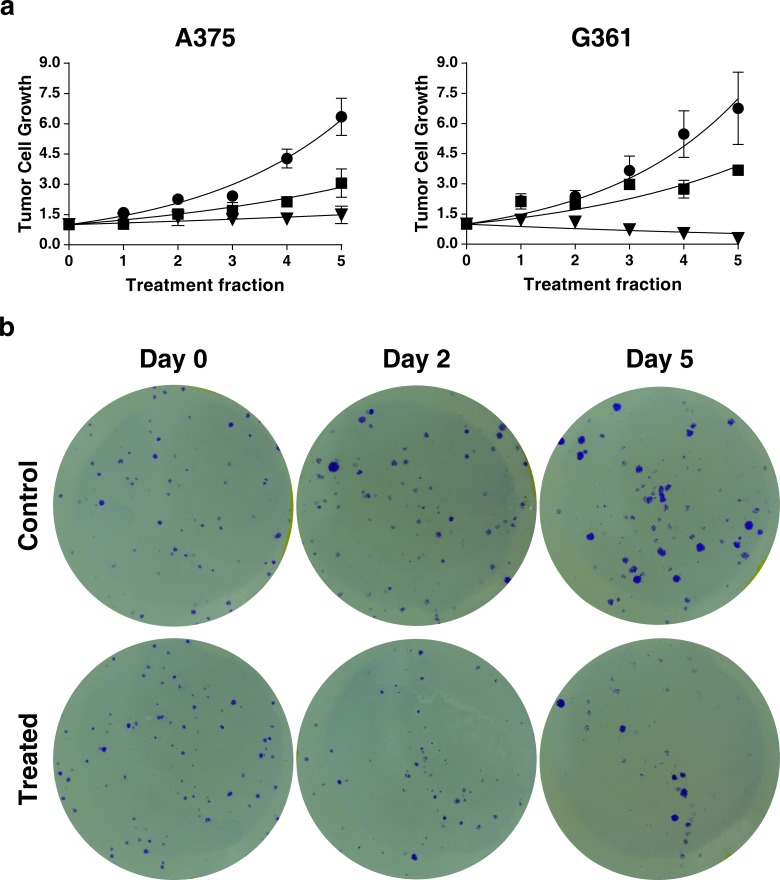
Superior panel **a**: the effect of RCM obtained 24h and 48h after 2 Gy irradiation of MSC used in the reiteration treatment on A375 and G361 tumor-cell lines. The initial size of the colonies was measured (point 0 in the time-course experiments) and successive treatments with RCM were applied for 5 days more. Control treatment is indicated as ●, treatment with 24h RCM as ▼ and treatment with 48h RCM as ■. The differences between the curves are statistically significant (*P* < 0.0001, *N* = 3). Inferior panel **b**: representative images of a time course experiment of the human melanoma cancer cell line G361, grown as colonies in a monolayer culture. Top figures: colonies without any treatment. Bottom figures: the effect of RCM 24h. Notice the dramatic differences in the number of colonies formed and theirsize, between both experiments.

To check if “radiation-induced bystander effects” happens in tumor cells when the MSCs have been irradiated, we first checked with a colony cell assay if factors secreted from irradiated MSCs into the RCM have an influence on tumor cell growth. Exposure of human melanoma tumor cell (G361 or A375) colonies (formed over 9 days) to conditioned medium from irradiated MSCs (RCM) revealed that RCM treatment of the formed colonies (RCM 24h or RCM 48h) produced a delay in the tumor-cell growth. Using the mathematics proposed by Steel [30] the cell loss rate derived from the treatment of G361 colonies with RCM 24h and RCM 48h are respectively 31.3 % and higher than 100%, and for A375 colonies are 42.5% (RCM 24h) and higher than 75% (RCM 48h). Graphs included in Figure 1 show that treatment with RCM, has a strong effect on the tumor-cell colonies, producing not only as low-down of the tumor cell growth, but even yielding a progressive reduction of the initial colony size. When the slope of these curves reaches a negative value (G361, RCM 48h) the rate of cell loss in the tumor is greater than the rate at which cells are being added to the tumor by mitosis. Thus, we can state that, in this case, the cell-loss rate promoted by therapy is greater than 100%. For further details see the supplementary information.

### The combined treatment of tumor cell colonies with radiation plus MSCs or irradiated MSCs shows additive/synergistic effects

**Figure 2:**
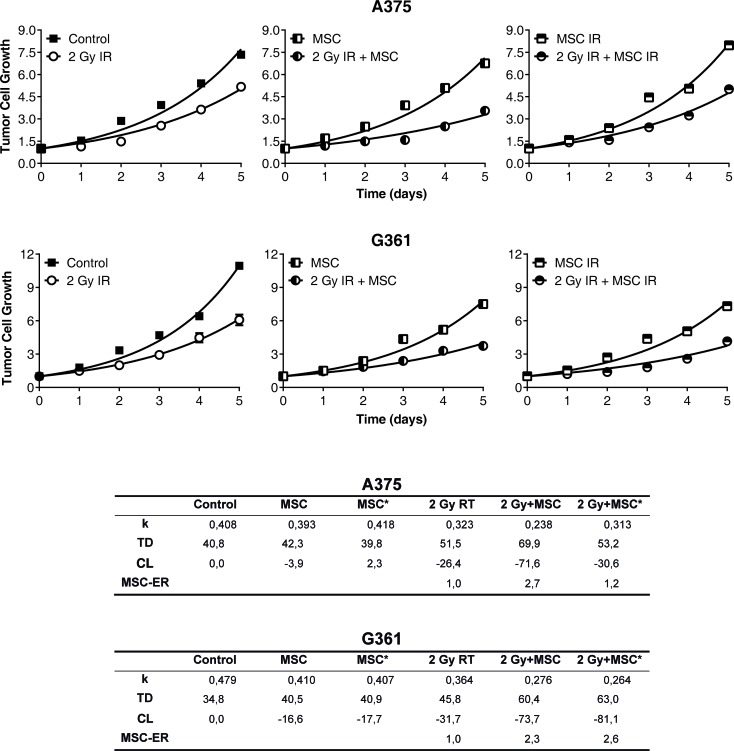
Growth of the A375 and G361 melanoma cancer-cell cell line in control conditions and after experimental treatments. First and second row: Panel 1: control condition vs irradiation with 2 Gy, Panel 2: co-culture of tumor cells with 2·10^5^MSCs or 2Gy irradiated tumor cell co-cultured with 2·10^5^MSCs, Panel 3: co-culture of tumor cells with 2·10^5^irradiated MSCs (MSC IR) or 2Gy irradiated tumor cell co-cultured with 2·10^5^irradiated MSCs (MSC IR). The initial size of the colonies was measured (point 0 in the time-course experiments) and their growth was monitored for 5 days more. Results presented as means ± SEM for triplicates of three independent assays. The experimental values were fitted using the non-linear regression method to an exponential equation and the differences between the curves are statistically significant (*P* < 0.0001). Parameters derived from the statistical treatment of these data, i.e. proportionality constant *k*, doubling time *T_D_*, and cell loss rate *C_L_* are summarized in the inferior panel.

After the treatments, the total area occupied by the colonies was measured on 5 consecutive days. Notably, quantification of the colony total area (plotted as the ratio to the value of the area occupied by colonies before irradiation and before the addition of MSC or MSC*) showed a significant growth delay in the colonies treated with radiation and MSCs or MSCs* compared to the growth of the same cells grown with radiation but without the addition of MSCs (Figure 2). The table contained in Figure 2 summarizes the results obtained after the fit of experimental points to an exponential equation. We have found that the presence of MSCs in co-culture with the tumor cells enhances the RT action. These results empirically prove that both tumor cell-colonies (A375 as well as G361) show a radiotherapy enhancement effect of the same magnitude when the treatment applied is RT + MSC. Therefore, we designed an experiment to check if this enhancement of radiotherapy effect is also measurable in an *in-vivo* situation.

### Radiation treatment of MSCs and tumor cells induced up-regulation of TRAIL and DKK3

**Figure 3:**
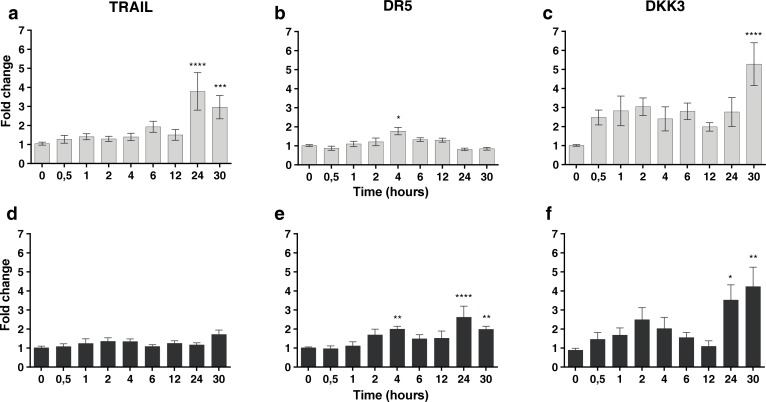
In superior panels, real-time quantitative RT-PCR for *TRAIL* and *DR5* and *DKK3* mRNA in MSCs treated with 2 Gy of ionizing radiation dose in a time-course experiment. In inferior panels real-time quantitative RT-PCR for *TRAIL* and *DR5* and *DKK3* mRNA in the MSCs treated with 6 Gy of ionizing radiation dose in a time-course experiment, the fold differences were calculated by the 2^−ΔΔCt^ method. In Y-axis values are means ± SEM for triplicates of three independent assays (*P < 0.05; **P < 0.01; ***P < 0.001 ****P < 0.0001). The results clearly demonstrate the up-regulation of *TRAIL* and *DKK3* mRNA at different times after irradiation.

To explore whether low doses of radiotherapy might induce the expression of different molecular factors in MSCs we performed quantitative PCR for a set of pre-selected candidatesinvolved in the induction of cell death [28]. Firstly, we observed that the expression of *TRAIL*, TRAIL receptor *DR5* and *DKK*3 mRNA were up-regulated in MSCs after treatment with 2 or 6 Gy (Figure 3). The results demonstrate the up-regulation of *TRAIL* at 24 and 30 hours, *DR5* after 4 hours and *DKK3* at 30 hours after irradiation. It is noteworthy that *DKK3* levels, after 2 Gy irradiation, are 5 times higher at 30 hours than basal levels. Survival for these cells after radiation treatment at 2 and 6 Gy was 25% and 2% respectively.

**Figure 4:**
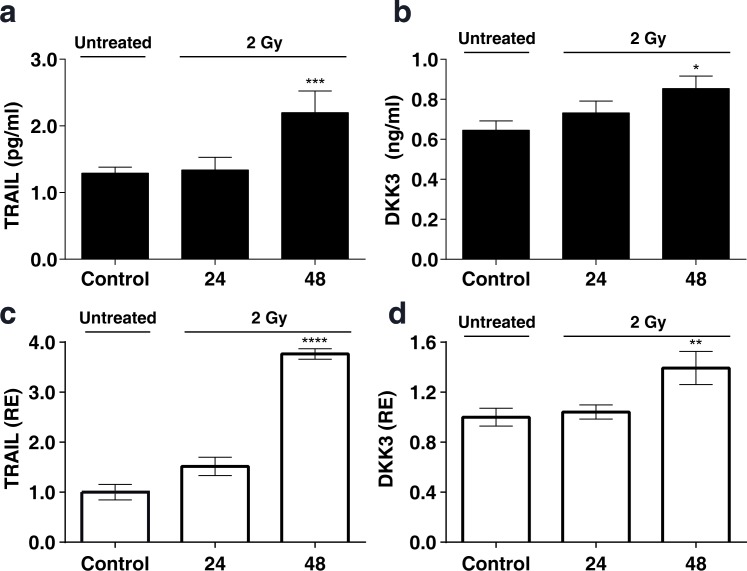
**a, b:** The levels of the TRAIL and DKK3 proteins measured by ELISA in the MSC culture medium, in a time-course, after stimulation with a 2 Gy irradiation after 24 hours and 48 hours. **c.**, **d.**: levels of TRAIL and DKK3 measured in the cells using the whole cell ELISA assay method (32) measuring the amount of membrane-tethered form of TRAIL on MSCs in basal (control) and treatment conditions 24 and 48h after irradiation. The Y-axis shows the relative results referred to as fold changes compared to control levels. (*P < 0.05; **P < 0.01; ***P < 0.001 ****P < 0.0001).

**Figure 5:**
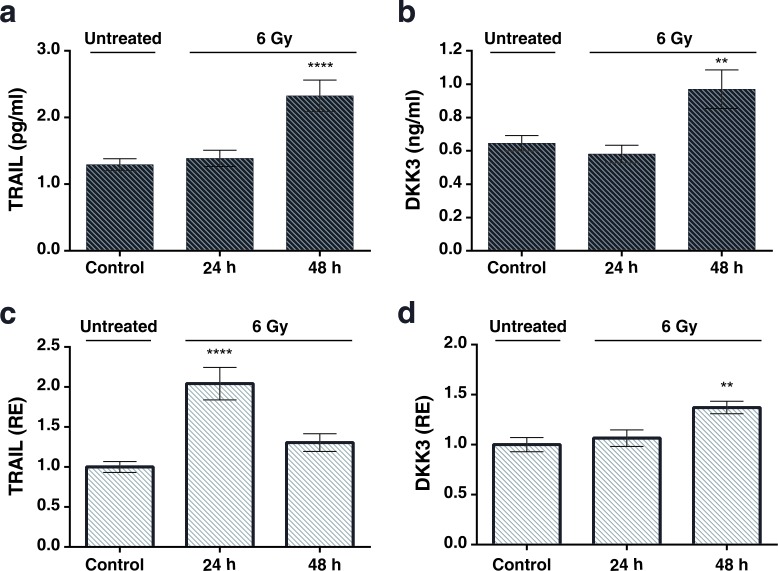
**a.** and **b.** Levels of the TRAIL and DKK3 proteins measured in in the cell-culture medium in a time-course evolution experiment after the stimulation of the MSCs with a 6 Gy irradiation. Results of unirradiated cells (control), 24h and 48 hours after irradiation. **c.** and **d.** Results of TRAIL and DKK3 levels by whole cell ELISA. Values are in pg/ml for enzyme-immune-assay of TRAIL, ng/ml for DKK3 and in relative units for the whole cell assay.

To determine whether the upregulation of TRAIL and DKK3 also occurs at the protein level, we performed ELISAs in conditioned medium from control and irradiated MSCs. The concentration of TRAIL and DKK3 in the culture medium, after 48 hours of cell treatment with 2 Gy and 6 Gy, was significantly higher than in the control condition (Figure 4a, 4b and Figure 5a, 5b) 1.29 ± 0.09 v.s. 2.20±0.32 pg/ml (*P* < 0.001) for TRAIL and 0.64 ± 0.05 and 0.85 ± 0.06 ng/ml for DKK3 for 2 Gy irradiation (*P* < 0.01). Using the whole cell assay method [31] (Figure 4c, 4d and Figure 5c, 5d) we observed the significant up-regulation of the transmembrane-tethered form of TRAIL on the MSCsat 48 h after 2 Gy treatment; in fact, the expression of TRAIL measured in the cells at 48h is more than 3 times greater than in the control (untreated) cells (*P* < 0.0001). We have also observed a significant increase in DKK3 in MSC whole cells after 48 h of 2 Gy and 6 Gy treatment (*P* < 0.01).

**Figure 6:**
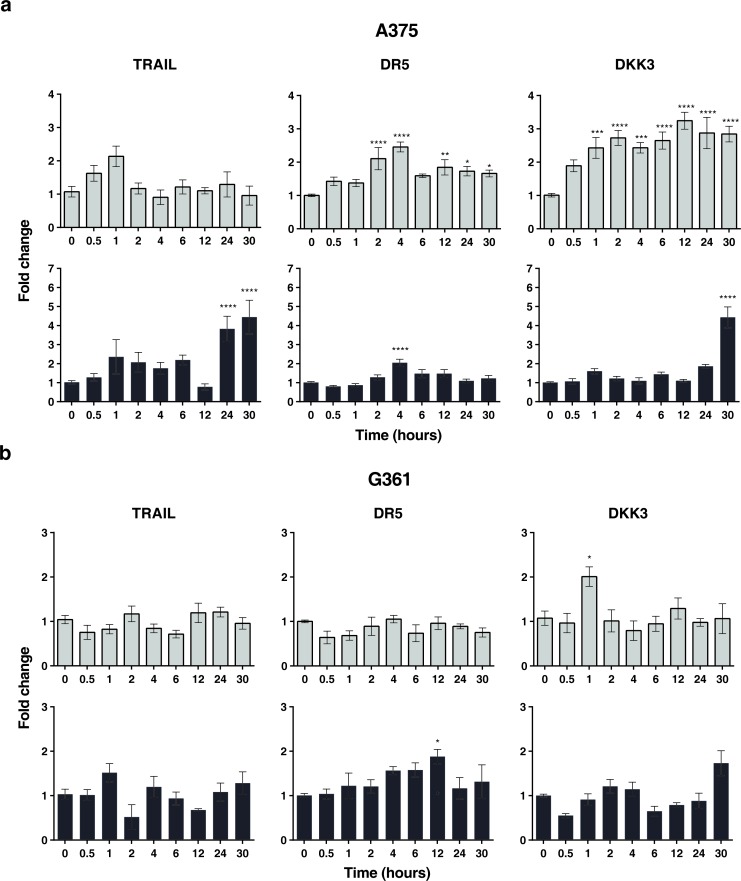
Real-time quantitative RT-PCR for *TRAIL* and *DR5* and *DKK3* mRNA in the A375 (**a**) and G361 (**b**) melanoma cell lines with the following treatment of a 2 Gy (light color) or 6 Gy (dark color) of ionizing radiation dose in a time-course experiment, the fold differences were calculated by the 2^−ΔΔCt^ method. In Y-axis values are means ± SEM for triplicates of 4 independent assays (*P < 0.05; **P < 0.01; ***P < 0.001 ****P < 0.0001). The most relevant difference is the up-regulation of *DR5* and *DKK3* in A375 and of *DKK3* in G361 which might have a radio-sensitizing effect on tumor cells. Of great interest is the absence of *DKK3* in this cell line. Up-regulation of *TRAIL* occurred at 24 and 30 hours for A375 and the up-regulation of *DR5* is evident for the two tumor cell lines assessed, although there are the same differences in the expression kinetics of this gene.

We also explored the expression changes of these factors upon radiation treatment in A375 and G361. Irradiation with 2 Gy led to the up-regulation of *DR5* and *DKK3* in A375 melanoma cells and up-regulation of *DKK3*in G361 (Figure 6). We also observed that after irradiation of the tumor cells with 6 Gy *TRAIL, DR5*and *DKK3* were up-regulated in A375 and we observed the up-regulation of *DR5* in G361 (Figure 6). Survival for these cells after radiation treatment at 2 Gy was 62 % for A375 and 60 % for G361.

Altogether, our results show that both MSCs and tumor cells respond to irradiation by enhancing the expression of the proteins TRAIL and DKK3 as well as of the TRAIL receptor DR5. These changes might have a radio-sensitizing effect on tumor cells.

### Tumor suppressor activity of MSCs *in vivo* and its combination with radiotherapy

**Figure 7:**
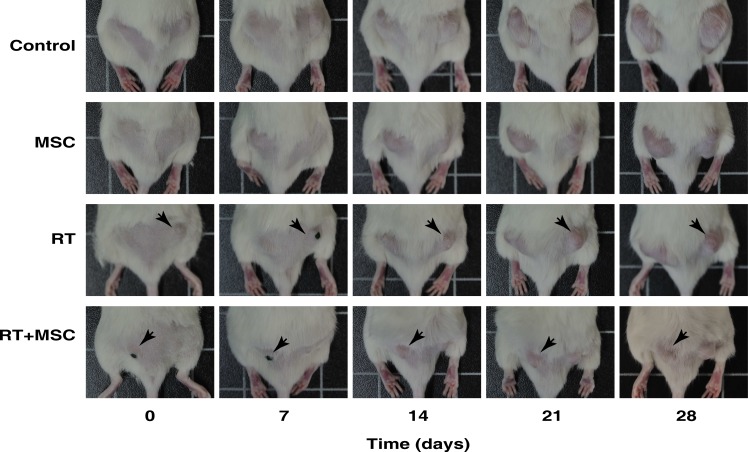
Representative images of mice inoculated with 1·106 G361 human melanoma cell line. Mice with a tumor size of approximately 65 mm3 were treated in different ways as indicated for 4 weeks after the start of the treatment Images of tumour growth were made at the beginning of the experiment (day 0) and at 7, 14, 21 and 28 days for the control group and after each type of treatment. The black dots that are visible in some mice are points to facilitate the tumor irradiation. In the RT group the tumor on the right side was irradiated (black arrows), whereas in the MSC + RT group the irradiated tumor was on the left side (black arrows). The tumors on the contralateral side were protected from irradiation and just affected by the bystander effect.

In order to elucidate the role of MSCs as a tumor-suppressor agent used alone or in combination with radiotherapy, we implanted tumor cells in both flanks of NOD/SCID mice to produce bilateral xenotumors. Mice treated with MSCs were injected intraperitoneally with 10^6^ MSCs once weekly during four sucessive weeks. Mice treated with RT (2 Gy) received radiation once weekly during 4 succesive weeks (Figure 7). In this set up, one flank with a tumor becomes irradiated and the other one remains naïve to radiotherapy and is only affected by the systemic bystander effect. Control experiments were performed to exclude direct radiation effects on the contra-lateral side (see supplementary information for the dosimetric procedure).

**Figure 8:**
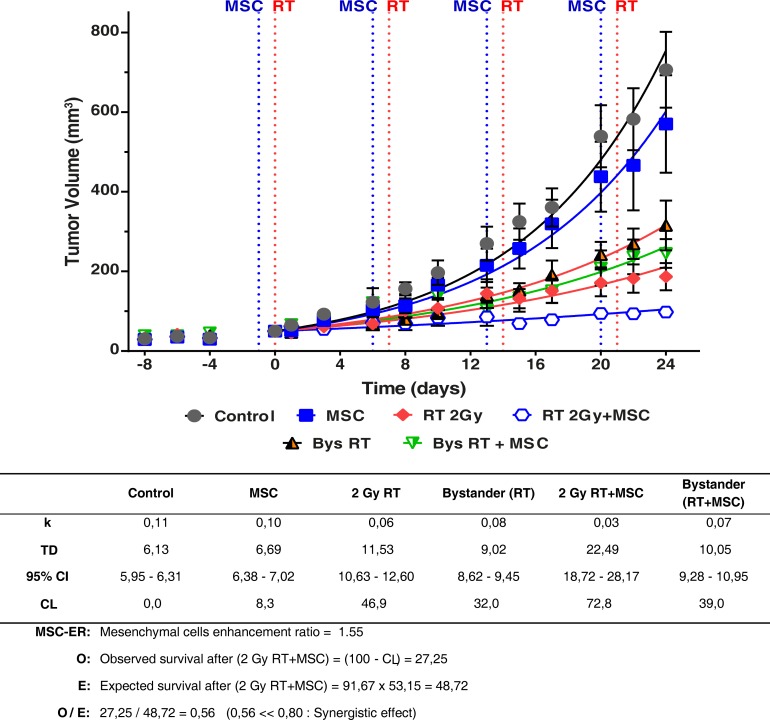
Tumor growth curves of the primary tumors and the contralateral tumors (bystander effect) in the G361 tumor model Animals were monitored regularly every 2-3 days, measuring two perpendicular diameters of each tumor for tumor growth. The treatment was repeated each week as indicated in the graph, for a total of four weeks. Experimental values were fitted using the non-linear regression method to an exponential equation. Tumor sizes are presented as mean ± SEM of at least 7 mice in each experiment. The effect of treatment on the unirradiated tumors (bystander effect) has been marked as Bys RT and Bys RT + MSC. The differences between the curves are statistically significant (*P* < 0.0001). Parameters derived from the statistical treatment of these data, i.e. proportionality constant *k*, doubling time *T_D_*, cell loss rate*C_L_* and mesenchymal cell enhancement ratio are summarized in the inferior panel. A statistically significant reduction in the tumor growth is observed in treatments with MSC and RT and the combination of RT+MSC give a synergistic result.

It is noteworthy that all the curves are different compared with the control tumor growth curve in Figure 8, (*P* < 0.0001) so, each *T_D_* value is characteristic for each curve. The table under the graph shows the values found for the growth rate (*k*) and doubling time (*T_D_*) in all the experimental situations studied. Notice the values of *T_D_* ranged from 6.69 to22.49 days. Using these values we have calculated the cell-loss rate that can be attributed to each treatment. The cell-loss factor, described and defined by Steel [30] was conceived as a measure of spontaneous cell loss, occurring when the tumor grows. In our case, cell loss is used to describe response to a therapy, and although the mathematics are the same, we used this parameter as a measure in which are included: (i) all the cell-death types, (ii) the situations in which the treatment produces a lengthening of the mean cell cycle duration, (iii) and cells that have a null or limited growth potential as a result of mis-repair of damage or because they have been involved in a differentiation process. According to this concept we can state that radiotherapy inhibited tumor growth with a cell loss rate of 46.9% per day compared to tumor growth in the control group (*P* < 0.0001) (Figures 7 and 8). This effect was enhanced by the addition of MSCs to the radiotherapy, leading to a nearly complete inhibition of tumor growth with a final volume of less than 15% of the tumor volume in the control experiment (*P* < 0.0001), whilst MSCs alone inhibited tumor growth with a cell loss rate of 8.3% (*P* = 0.0013). Altogether, these data demonstrate that MSCs potentiate the radiotherapy effect when infused into tumor-bearing mice.

### *In vivo* bystander effect of irradiation and MSC therapy

Of interest is the bystander effect of the radiotherapy on the tumor of the contra-lateral side, which by itself led to an inhibition of tumor growth corresponding to a cell-loss rate of 32% compared to the tumor growth in the control group (*P* < 0.0001). Tumors from the non-irradiated flank, thus exposed to the bystander effect after RT + MSC treatment, showed a further inhibition of tumor growth with a rate of cell loss of 39% compared to the tumor growth under control conditions (*P* < 0.0001). The differences between the tumor growth curves of bystander tumors, in the mice with tumors treated with RT 2Gy and RT 2 Gy + MSC, are also statistically significant (*P* = 0.0154). Moreover, at the end of the experiment the tumor volumes in each of the experimental groups showed a volume reduction, compared to the control group, 19% for MSCs, 74% for RT, 55%for bystander RT, 86% for RT+MSC and 65% for bystander RT+MSC (Figure 8).

The treatment of tumors with radiotherapy produced a cell-loss rate [30] of 46.9%; the complementary value (SF = 100-C_L_) of the surviving percentage was 53.1%. The same parameter measured for treatment with MSCs administrated once a week for 4 weeks was 8.3 % (SF = 91.7%) compared to the control experiment. Finally, the cell loss rate for the combination of RT and MSC reached 72.8 %. Supposing that the effects for each of the treatments (RT and MSC) are independent [32], the expected value (E) for the surviving fraction after the treatment with RT + MSC is: E = 46.9 · 91.7 = 48.7%. On the other hand the observed value for the surviving fraction after RT+MSC is O = 27.2% (table included in Figure 8). Using both data we have calculated the ratio O/E = 0.56, which is a strong indicator of the synergistic effect between RT and MSC when they are applied together for tumor treatment in this model. These results demonstrate the potentiation of the bystander effect by the MSCs used together with radiotherapy.

### Histopathological and immunohistochemical studies show the inhibition of proliferation activity in the tumors treated

**Figure 9:**
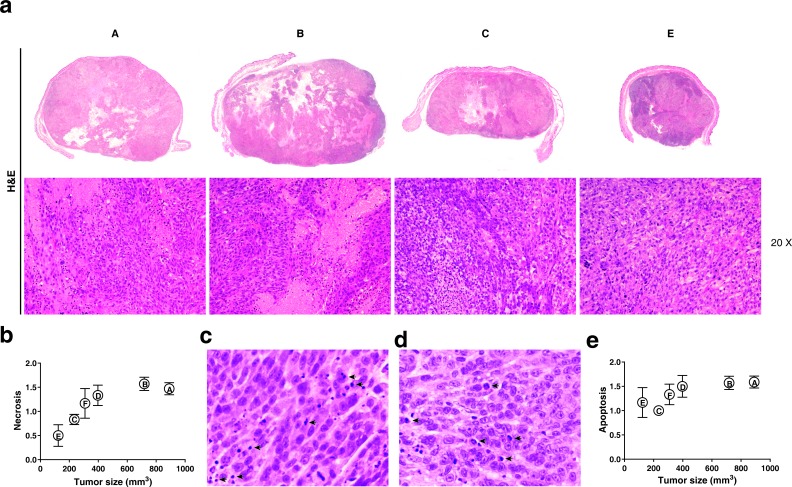
**a.** Representative panoramicimages and photomicrographs of melanoma G361 cell line xenograft on mice. A: Control; B: MSC: C: RT(2Gy) and E: RT(2Gy)+MSC. Images corresponding at D and F groups are not included. Note large areas of necrosis in control and MSC groups, hematoxylin-eosin, original magnification x20 to obtain data of mitotic index, score of necrosis and apoptosis. **b.** The study of the association between the score obtained for necrotic area on the tumor demonstrates a marked trend with the tumor volume in the smaller tumors but this relationship lacks statistical significance when tumors are larger than 400 μm. **c.** and **d.** Representative photomicrographs of H&E where it is possible to identify apoptotic cells (black arrows). **e.** We have no found association between the apoptotic score and treatment nor the tumor size.

To identify whether the reduction in tumor volume was due to reduced proliferation and/or cell death of tumor cells, we performed histopathological and immunohistochemical analyses at the end of the experiment. Using H&E stainings we analyzed the mitotic index [33] of the tumors in the different experimental treatment groups, as well as the presence of apoptosis and necrosis. (Figure 9a). The normalization of the necrotic area compared to the same parameter in the control tumor (Figure 9b) suggests that the increase of the necrotic area, within the tumors, is related with the increase of tumor size for tumors, whose volume ranged between 100 and 400 mm^3^ and indicates that the treated tumors have a lesser proportion of necrotic area than tumors placed in the contra-lateral side which are just affected by the bystander effect, although the results are not statistically significant (*P* > 0.05). Evaluation of apoptotic cells by morphologic examination i.e. significant DNA hyperchromicity, chromatin condensation and karyorrhexis (nuclear fragmentation) of tumor sections revealed that apoptosis did not differ between the groups (Figure 9c, 9d and 9e), which is consistent with the view that apoptosis is not as dominant a process in cell loss from tumors [34] as has sometimes been claimed.

**Figure 10:**
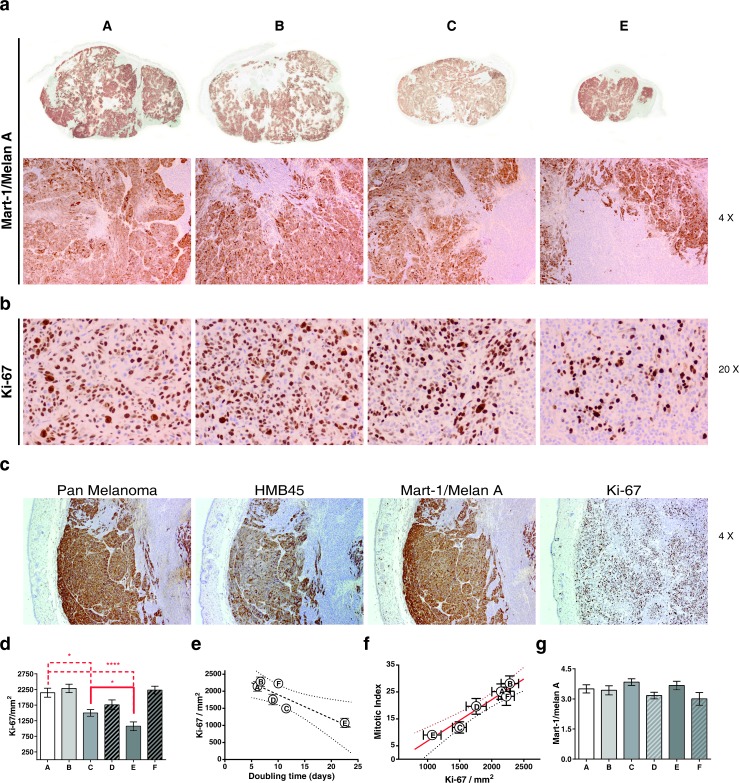
A: Control; B: MSC: C: RT(2Gy) and E: RT(2Gy)+MSC. **a.** Photomicrographs of Mart-1/melan A, immunohistochemical expression of the melanoma G361 cell line xenograft on mice. First row, panoramic view. Second row, note the two types of cell populations, negative and positive, for Mart-1/MelanA in all groups. **b.** Photomicrographs of Ki-67 immunohistochemical expression at the end of the trial in G361 xenotumors treated indifferent ways. The nuclear Ki-67 expression was lesser in RT and in RT(2Gy)+MSC groups. **c.** Similar immunohistochemical expression of three melanoma markers in G361 cell line xenograft on mice from control group. Note two types of cell populations for several markers of differentiation (micropolymer peroxidase-conjugated, original magnification x20). **d.** Therelationship between the values of MI measured with hematoxiline-eoxine and with Ki-67 staining. **e.** Experimental points fitted to a straight line (R^2^ = 0.939). **f.** Relationship between the MI and Ki-67 (*P* = 0.0014). **g.** We have no found association between the expression of Mart-1/Melan A assessed by score and the tumor size.

**Figure 11:**
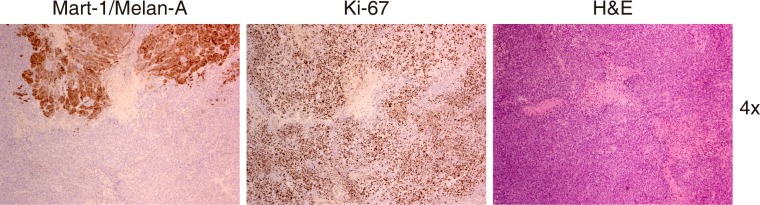
Representative images obtained from the same tumor area that is mainly negative for the expression of Mart-1/Melan A. The morphology of these cells is somewhat different to the use aspect of the melanoma cells with pale cytoplasm, greater nuclei and evident nucleoli. These cells are smaller, with atypical mitosis but the number of cells in proliferative state is similar to the found in areas with positive expression of Mart-1/Melan A and are clearly different tothose that belong to the stroma around the vases, these are smaller and their distribution is not in a cluster. Taking all these characteristic and the negative expression of Mart-1/Melan A into account, we believe that these are less differentiated melanic cells.

To analyze the proportion of melanoma tumor cells within the tumor mass in control or in treated tumors, Mart1/MelanA (a marker for cells with melanocytic differentiation) stainings were performed (Figure 10 and Figure 11). These stainings clearly show the existence of a double tumor cell population within the tumors that can be distinguished by differential expressions of the melanoma markers Pan-Melanoma, HMB45 and Mart-1/MelanA, which may indicate the presence of two states of differentiation of the tumor cells (Figure 10c). After treatment we have not found differences when quantifying the percentage of area ocupied by Mart-1/MelanA+ cells in the different groups (Figure 10g), indicating that all the treatments affected both populations in the same way. *Vice versa*, proliferation also occurred at the same level in the tumor cells Mark1/Melan A positive as in the negative (Figure 10c, Ki-67 image). We finally analyzed tumor proliferation by quantifying Ki-67+ cells. Mititoc index (MI) and Ki-67 staining were closely related (Figure 10f). Consistent with the results obtained in the study of tumor growth kinetic, the tumor proliferation was reduced upon irradiation when compared to control treated tumors (Figure 10b, 10d, 10e). Moreover, the addition of MSCs to the radiotherapy treatment, led to a reduced proliferation in the tumor compared with the proliferation level that existed after RT alone (*P* = 0.0356, Figure 10d), supporting our previous statement of a sinergistic effect when both treatments, RT + MSC, are combined.

## DISCUSSION

In this study we have shown that MSC-derived radiation conditioned medium (RCM) is cytotoxic for tumor cells and that the combined treatment with radiation and MSCs can produce synergistic cytotoxic effects on tumor cells, both *in vitro* and *in vivo*. This offers a potential new treatment option in advanced tumors.

*In vivo*, the decreased number of Ki-67^+^ cells within the tumors treated with RT or with RT+MSC versus control tumors (*P* = 0.0162 and *P* = 0.0004, respectively, Figure 10c, 10d), as well as in tumors treated with RT + MSCs versus RT alone (*P* = 0.0356), supports the synergistic effect between RT and MSCs. As the doubling time values are inversely related with the corresponding Ki-67 values (*P* = 0.0223, Figure 10e) we conclude that the major mechanisms underlying the delay of tumor growth are the increase of cell loss rate, and the decrease in the tumor-cell proliferation activity.

*In vitro* we show that irradiation of MSCs stimulates the secretion of TRAIL and DKK3 molecules that, amongst others, may promote tumor cell loss through the bystander effect, affecting the primary tumor together with radiation and additionally having a distant bystander systemic anti-tumor action.

The amount of apoptotic cells within the *in vivo* tumor does not seem to be related to the tumor size (measured at the moment of tumor excision) nor to the treatment modality, probably because apoptosis is not a predominant cause of cell death after radiation therapy [34]. Accumulating evidence indicates that, if apoptosis is disabled, cancer cells may be eliminated by other mechanisms. Interestingly, TRAIL and DKK3, apart from inducing tumor-cell apoptosis [28], have also been shown to inhibit proliferation in several tumor and non-tumor cell types [35, 36]. Thus, it is possible that the observed decreased in Ki67^+^ cells in RT+MSC treated tumors is mediated by MSC-derived TRAIL and DKK3. In fact radiation has been demonstrated, in different cell models and circumstances, to induce all the various forms of cell death [37]. Finally, as the necrotic area seems to be related to the size of the tumor, it does not appear to be the underlying mechanism for therapy response and its contribution remains unclear.

Our data suggest that a combined treatment with radiation and MSCs might be used as: (1) a strategy for the radio-sensitization of tumors and (2) a systemic tool for treating advanced cases of cancer

### A strategy for radio-sensitization of tumors

We have evaluated to what extent MSCs can enhance the therapeutic effect of radiation therapy, both *in vitro* as well as *in vivo.* From a theoretical point of view this can be described as the “mesenchymal cell enhancement ratio” (MSC-ER) and we can state that the addition of MSC treatment to radiotherapy is a potential tool for increasing the efficacy of radiotherapy through the expansion of the compartment of ‘activated cell’ [15] (for further details see supplementary materials). The resulting value of MSC-enhancement ratio of radiotherapy is 1.55 for in the *in vivo* experiments.

The clinical application of MSCs continues to significantly increase [38], demonstrating its safety and efficacy.

### A systemic tool to treat advanced cases of cancer

Radiotherapy is one of the most effective treatments for cancer, with more than half of all cancer patients estimated to receive radiotherapy at some point during their treatment. Here we show that the activation of MSCs with a 2 Gy low-LET radiation dose is enough to induce up-regulation of *TRAIL* and *DKK3* and, as a consequence, an increase in soluble TRAIL and DKK3 is detected in conditioned medium from the MSC treated with radiation. *DKK3* is significantly down-regulated in a broad range of cancer cell types [39]. Therefore, the release of DKK3 by the MSCs, when activated with radiation, is a useful approach for the generation of anti-tumor immune responses in malignant cells.

Ionizing radiation causes DNA damage via free radicals and direct ionizations [3, 4, 40-42], however in most cases cells die only after attempting mitosis, during which the improperly repaired DNA damage causes mitotic catastrophe, which subsequently leads to cell death. Our *in vitro* data shows that conditoned medium from radiation-activated MSCs are able to reduce tumor-cell colony formation. Thus, we believe that the crosstalk between cell death and proliferation inhibition pathways [43] is one possible rationale of how diverse cytotoxic agents can exhibit tumor-selective killing and how radiation conditionated medium acts on the cancer cells. Apart from our findings showing that TRAIL and DKK3 become upregulated and secreted upon irradiation, it has been described that other factors secreted by cells, such as TGF-β, interleukin-8, IL-15 or TNF-α, may be involved in the propagation of the bystander effect [17, 18, 44-50].

Interestingly our results also show a greater increase in the transmembrane TRAIL in MSCs after 2 Gy radiotherapy than in the TRAIL secreted (Figure 3). DNA, RNA [28] or exosomes released from the cells might increase interactions of tumor cells and MSCs and enhance secreted microRNAs which were shown to have an antiproliferative action on the tumor cells [51, 52], and thereby increase the effectiveness of MSCs in suppressing tumor progression in mice.

After the study of all the results included in this paper, we believe that both TRAIL and DKK3 are involved in the bystander effect observed. In fact, our data shows an association but does not prove cause and effect. There may be cause and effect, but that still has to be demonstrated. We are also convinced that, together with TRAIL and DKK, other molecules produced by the MSC after irradiation and, perhaps, included in microvesicules or exosomes, maybe important in this effect. Clearly this is an area for further work.

In our *in vivo* experiments the combination of MSC cell therapy plus radiotherapy in NOD/SCID mice significantly reduced the size of the established tumors, both in the primary-directly irradiated tumor as well as in the distant non-irradiated tumor.

## CONCLUSIONS

We believe that MSCs could be used as a therapy against cancer as they are able to preferentially home onto tumors and when pre-activated, or when activated directly with radiation *in vivo*, secrete cytoquines and tumor suppressor proteins which produce a dramatic improvement in the biological effect of ionizing radiation on tumors.

These radiation activated cells could be considered as “medicinal cells” due to their tropism and immunomodulatory properties, and since after activation with radiotherapy and included in the tumor burden, may serve as site-regulated “drugstores”.

## MATERIALS AND METHODS

### Cell lines and culture

Umbilical cord stromal stem cells (MSCs) were prepared and cultured as previously described [21]. Tumor cell lines A375 and G361 were cultured as previously described [12]. The medium was replaced every 2-3 days and once 70-80% confluence was reached the cells were sub-cultured.

### Experimental irradiation of cell lines and preparation of radiation conditioned medium (RCM)

Exponentially grown MSC cells at 70% confluence were irradiated (2 or 6 Gy) at room temperature at a dose-rate of 2 Gy/min using a ^137^Cs irradiator. The culture medium was then replaced with fresh culture medium 24 hours before irradiating the cells, collected and filtered at 24 hours (RCM 24 h) or 48 hours (RCM 48 h) after irradiation to produce the MSC radiation conditioned medium used in the transfer medium assays.

### Cell treatment with radiation conditioned medium and the colony cell assay

In all the clonogenic assays A375 and G361 cells were seeded (2·10^2^ cells per well) and were allowed to form colonies for 9 days. The colonies were then treated for 24 hours with either fresh medium or RCM 24h or RCM 48h. The treatment was repeated 5 times with a 24-hour interval on the same tumor-cell colonies.

24 h after each treatment we fixed and stained three wells of each condition each day. The colonies were fixed with 70% ethanol and stained with crystal violet. The colony count was performed on photographed wells and analysed with the software ImageJ. The total area occupied by the colonies was quantified and compared with the colonies from the untreated condition. The results of these experiments have been fitted to an exponential equation to calculate the duplication time value (*T_D_*) of the colonies for each set of data.

### Co-cultures of UCSSC-MSC and tumor colonies

We then co-cultured MSC cells with tumor colonies to address their effect on tumor growth kinetics. Tumor cells were seeded as before. Subsequently, tumor-formed colonies were treated with a dose of 2 Gy of irradiation and co-cultured with irradiated, non-irradiated MSC or left untreated for 6 more days. Each day three wells of each condition were fixed with 70% ethanol and stained with violet crystal. The parameters of growth of control experiment as well as of treatment colonies for the whole treatment period were then calculated. For further detail see the supplementary information.

### RNA extraction and quantitative real-time PCR

RNA from MSC, A375 and G361 cell lines was extracted using TRIzol^®^ Plus RNA Purification Kit (Life Technologies). A375, G361 and MSC were exposed to 2 or 6 Gy of gamma radiation and total RNA was extracted after 0, 0.5, 1, 2, 4, 6, 12, 24 and 30 hours. Primers were designed for the relative quantitation of the gene expression of *TRAIL*, TRAIL receptor *DR5* and *DKK3* mRNA expression in each cell line. For further details see the supplementary information.

Approximately 1 μg of total RNA extracted from cultured cells was used to synthesize double-stranded complementary DNA (cDNA) by reverse transcription using iScript^TM^ cDNA Synthesis Kit (Bio-rad), following the manufacturer's instructions. Complementary DNA was analysed by real-time PCR using iQ SYBR Green Supermix (Bio-Rad).

### ELISA of UCSSC-MSC radiation conditioned medium (RCM)

Culture medium from MSC irradiated at 2 or 6 Gy was collected 4, 6, 12, 24 and 30 hours after irradiation. TRAIL and DKK3 concentration on MSC-RCM was determined by sandwich ELISA using a human CD253 (TRAIL) (OptEIA, BD) and a human DKK3 (DuoSet ELISA development system, R&D), respectively, following the manufacturer's instructions.

### ELISA of umbilical cord MSC whole cells

The levels of TRAIL and DKK3 protein expression in whole cells were quantified by ELISA as described elsewhere [31]. For further details see supplementary information.

### Mice, tumor xenographs, *in vivo* tumor growth and treatments

In our experiments 7- to 9-week-old NOD/SCID mice were engrafted with cells of the human cancer line G361. Four groups of eight mice were each treated with radiotherapy, MSC therapy, radiotherapy plus MSC therapy, or left untreated (control).

These studies were performed in strict accordance with the recommendations of the Guide for the Care and Use of Laboratory Animals of the Bioethical Committee of Granada University, and the protocol was approved by the Committee on the Ethics of Animal Experiments of the CSIC. All surgery was performed under isoflurano anesthesia, or ketamine when necessary, and every effort was made to minimize the suffering of the mice.

Cells were inoculated into dorsal skin folds of NOD/SCID mice (1·10^6^ cells in 0.1 ml of saline serum on each hind leg). The mice were monitored regularly every 2-3 days, measuring two perpendicular diameters of each tumor for tumor growth. For further detail see the supplementary information.

**Radiotherapy Group**: One group (8 mice) with a tumor on each hind leg was anesthetized with Ketamine and only one of the tumors was treated with a dose of 2 Gy. Ionizing radiation was delivered by X-Ray TUBE (YXLON, model Y, Tu 320-D03). The mice tumors were placed directely under a 10 mm diameter hole in a 8 mm thick steel sheet. The contra-lateral tumor, as well as the rest of the body of each mouse, was protected by the steel sheet (for further details see supplementary information). The treatment was repeated each week, for a total of four weeks. After the last dose the animals were left to rest for 3-4 days before the experiments were finalized.

**Control Group**: One group (8 mice) with tumors on each leg was handled in exactly the same way as the irradiated and MSC injected mice, although the group did not receive either radiation or MSC therapy.

**MSC therapy groups**: Two groups (8 mice in each group) with tumors larger than 64 mm^3^ were treated with a repeated intraperitoneal administration of 10^6^ MSC administrated every Monday for 4 successive weeks. The day after this cellular treatment, one of the groups (8 mice) was randomly selected to have one of their tumors irradiated each week as in the experiment described previously. The other group was monitored and treated with repeated injections of MSC every week for 4 weeks.

Throughout the successive intervals between treatments, all the mice included in the control, radiotherapy, MSC therapy and radiotherapy plus MSC therapy, were monitored to measure the tumor sizes and follow the tumor growth as a time and treatment function.

### Tumor growth calculations

From the fit of the experimental data to for the growth of tumors as a function of time to an exponential equation we can obtain the value of the slope and, using this, the values for the duplication time (*T_D_*). (For further detail see the supplementary information). Thus, cell loss rate (*C_L_*) derived from each treatment was calculated using the (*T_D_*) values and the equation proposed by Steel [30]. The surviving fraction after each treatment (*S_F_*) is: (*S_F_* = 1 − *C_L_*). The theoretical expected value (E) for survival fraction after the combination of treatments RT + MSC was calculated by multiplication of the *S_F_* after RT times *S_F_* after MSC. The actual effect on cell proliferation when both treatments are combined (RT+MSC) is the observed value (O) which is calculated from the time course experiment. The additive model [32] predicts whether the combined effects of two treatments are synergistic when the ratio O/E < 0.8; additive when O/E = 0.8-1.2; or sub-additive when O/E > 1.2.

### Histopathological and immunohistochemical studies

At the end of the experiment, the dissected tumors were immediately fixed in 10% buffered formalin for 48 h, and then embedded in paraffin, and 4μm sections were dewaxed, hydrated, and stained using the hematoxylin-eosin technique. On these slides we determined the mitotic index [33], the Ki-67 reactivity, the necrotic areas, the amount of apoptotic cells outside the necrotic areas, and the percentege of area ocupied by melanoma cancer cells that expressed the Mart-1/MelanA antigen. For further details see the supplementary materials.

## SUPPLEMENTARY MATERIAL FIGURE



## References

[1] Steel GG, McMillan TJ, Peacock JH (1989). The 5Rs of radiobiology. International journal of radiation biology.

[2] Peacock JH, de Almodovar MR, McMillan TJ, Steel GG (1992). The nature of the initial slope of radiation cell survival curves. BJR Suppl.

[3] Ruiz de Almodovar JM, Steel GG, Whitaker SJ, McMillan TJ (1994). A comparison of methods for calculating DNA double-strand break induction frequency in mammalian cells by pulsed-field gel electrophoresis. International journal of radiation biology.

[4] Ruiz de Almodovar JM, Nunez MI, McMillan TJ, Olea N, Mort C, Villalobos M, Pedraza V, Steel GG (1994). Initial radiation-induced DNA damage in human tumour cell lines: a correlation with intrinsic cellular radiosensitivity. British journal of cancer.

[5] Ruiz de Almodovar JM, Guirado D, Nunez MI, Lopez E, Guerrero R, Valenzuela MT, Villalobos M, del Moral R (2002). Individualization of radiotherapy in breast cancer patients: possible usefulness of a DNA damage assay to measure normal cell radiosensitivity. Radiotherapy and oncology: journal of the European Society for Therapeutic Radiology and Oncology.

[6] Lopez E, Guerrero R, Nunez MI, del Moral R, Villalobos M, Martinez-Galan J, Valenzuela MT, Munoz-Gamez JA, Oliver FJ, Martin-Oliva D, Ruiz de Almodovar JM (2005). Early and late skin reactions to radiotherapy for breast cancer and their correlation with radiation-induced DNA damage in lymphocytes. Breast cancer research: BCR.

[7] Guirado D, Ruiz de Almodovar JM (2003). Prediction of normal tissue response and individualization of doses in radiotherapy. Physics in medicine and biology.

[8] Mothersill C, Seymour C (1997). Medium from irradiated human epithelial cells but not human fibroblasts reduces the clonogenic survival of unirradiated cells. International journal of radiation biology.

[9] Mothersill C, Seymour CB (2004). Radiation-induced bystander effects—implications for cancer. Nat Rev Cancer.

[10] Formenti SC, Demaria S (2009). Systemic effects of local radiotherapy. Lancet Oncol.

[11] Azzam EI, de Toledo SM, Little JB (2001). Direct evidence for the participation of gap junction-mediated intercellular communication in the transmission of damage signals from alpha -particle irradiated to nonirradiated cells. Proceedings of the National Academy of Sciences of the United States of America.

[12] Gomez-Millan J, Katz IS, Farias Vde A, Linares-Fernandez JL, Lopez-Penalver J, Ortiz-Ferron G, Ruiz-Ruiz C, Oliver FJ, Ruiz de Almodovar JM (2012). The importance of bystander effects in radiation therapy in melanoma skin-cancer cells and umbilical-cord stromal stem cells. Radiotherapy and oncology: journal of the European Society for Therapeutic Radiology and Oncology.

[13] Begg AC, Stewart FA, Vens C (2011). Strategies to improve radiotherapy with targeted drugs. Nat Rev Cancer.

[14] Lambin P, van Stiphout RG, Starmans MH, Rios-Velazquez E, Nalbantov G, Aerts HJ, Roelofs E, van Elmpt W, Boutros PC, Granone P, Valentini V, Begg AC, De Ruysscher D, Dekker A (2013). Predicting outcomes in radiation oncology—multifactorial decision support systems. Nat Rev Clin Oncol.

[15] Lara PC, Lopez-Penalver JJ, Farias VD, Ruiz-Ruiz MC, Oliver FJ, Ruiz de Almodovar JM (2014). Direct and bystander radiation effects: A biophysical model and clinical perspectives. Cancer letters.

[16] Prise KM, O'Sullivan JM (2009). Radiation-induced bystander signalling in cancer therapy. Nat Rev Cancer.

[17] Azzam EI, de Toledo SM, Little JB (2003). Oxidative metabolism, gap junctions and the ionizing radiation-induced bystander effect. Oncogene.

[18] Dickey JS, Baird BJ, Redon CE, Sokolov MV, Sedelnikova OA, Bonner WM (2009). Intercellular communication of cellular stress monitored by gamma-H2AX induction. Carcinogenesis.

[19] Goodhead DT (2010). New radiobiological, radiation risk and radiation protection paradigms. Mutat Res.

[20] Pinar B, Lara PC, Lloret M, Bordon E, Nunez MI, Villalobos M, Guerrero R, Luna JD, Ruiz de Almodovar JM (2007). Radiation-induced DNA damage as a predictor of long-term toxicity in locally advanced breast cancer patients treated with high-dose hyperfractionated radical radiotherapy. Radiat Res.

[21] Farias VA, Linares-Fernandez JL, Penalver JL, Paya Colmenero JA, Ferron GO, Duran EL, Fernandez RM, Olivares EG, O'Valle F, Puertas A, Oliver FJ, Ruiz de Almodovar JM (2011). Human umbilical cord stromal stem cell express CD10 and exert contractile properties. Placenta.

[22] Kim SM, Oh JH, Park SA, Ryu CH, Lim JY, Kim DS, Chang JW, Oh W, Jeun SS (2010). Irradiation enhances the tumor tropism and therapeutic potential of tumor necrosis factor-related apoptosis-inducing ligand-secreting human umbilical cord blood-derived mesenchymal stem cells in glioma therapy. Stem cells.

[23] Bergfeld SA, Blavier L, Declerck YA (2014). Bone marrow-derived mesenchymal stromal cells promote survival and drug resistance in tumor cells. Molecular cancer therapeutics.

[24] Yagi H, Kitagawa Y (2013). The role of mesenchymal stem cells in cancer development. Frontiers in genetics.

[25] Green DR (2010). Cell competition: pirates on the tangled bank. Cell stem cell.

[26] Caplan AI, Dennis JE (2006). Mesenchymal stem cells as trophic mediators. Journal of cellular biochemistry.

[27] Kanehira M, Kikuchi T, Santoso A, Tode N, Hirano T, Ohkouchi S, Tamada T, Sugiura H, Harigae H, Ichinose M (2014). Human marrow stromal cells downsize the stem cell fraction of lung cancers by fibroblast growth factor 10. Molecular and cellular biology.

[28] Lee RH, Yoon N, Reneau JC, Prockop DJ (2012). Preactivation of human MSCs with TNF-alpha enhances tumor-suppressive activity. Cell stem cell.

[29] Ranganath SH, Levy O, Inamdar MS, Karp JM (2012). Harnessing the mesenchymal stem cell secretome for the treatment of cardiovascular disease. Cell stem cell.

[30] Steel GG (1967). Cell loss as a factor in the growth rate of human tumours. European journal of cancer.

[31] Siles E, Villalobos M, Valenzuela MT, Nunez MI, Gordon A, McMillan TJ, Pedraza V, Ruiz de Almodovar JM (1996). Relationship between p53 status and radiosensitivity in human tumour cell lines. British journal of cancer.

[32] Valeriote F, Lin H (1975). Synergistic interaction of anticancer agents: a cellular perspective. Cancer chemotherapy reports Part 1.

[33] Evans AT, Blessing K, Orrell JM, Grant A (1992). Mitotic indices, anti-PCNA immunostaining, and AgNORs in thick cutaneous melanomas displaying paradoxical behaviour. The Journal of pathology.

[34] Steel GG (2001). The case against apoptosis. Acta oncologica.

[35] Song K, Chen Y, Goke R, Wilmen A, Seidel C, Goke A, Hilliard B, Chen Y (2000). Tumor necrosis factor-related apoptosis-inducing ligand (TRAIL) is an inhibitor of autoimmune inflammation and cell cycle progression. The Journal of experimental medicine.

[36] Kawano Y, Kitaoka M, Hamada Y, Walker MM, Waxman J, Kypta RM (2006). Regulation of prostate cell growth and morphogenesis by Dickkopf-3. Oncogene.

[37] Wouters BG, Joiner MC, van der Kogel A (2009). Basic Clinical Radiobiology.

[38] Maziarz RT, Devos T, Bachier CR, Goldstein SC, Leis JF, Devine SM, Meyers G, Gajewski JL, Maertens J, Deans RJ, Van't Hof W, Lazarus HM (2015). Single and multiple dose MultiStem (multipotent adult progenitor cell) therapy prophylaxis of acute graft-versus-host disease in myeloablative allogeneic hematopoietic cell transplantation: a phase 1 trial. Biology of blood and marrow transplantation: journal of the American Society for Blood and Marrow Transplantation.

[39] Tsuji T, Miyazaki M, Sakaguchi M, Inoue Y, Namba M (2000). A REIC gene shows down-regulation in human immortalized cells and human tumor-derived cell lines. Biochemical and biophysical research communications.

[40] Ward JF, Evans JW, Limoli CL, Calabro-Jones PM (1987). Radiation and hydrogen peroxide induced free radical damage to DNA. The British journal of cancer Supplement.

[41] Ward JF (1990). The yield of DNA double-strand breaks produced intracellularly by ionizing radiation: a review. International journal of radiation biology.

[42] Suzuki-Karasaki M, Ochiai T, Suzuki-Karasaki Y (2014). Crosstalk between mitochondrial ROS and depolarization in the potentiation of TRAIL-induced apoptosis in human tumor cells. International journal of oncology.

[43] Tait SW, Green DR (2010). Mitochondria and cell death: outer membrane permeabilization and beyond. Nature reviews Molecular cell biology.

[44] Sasportas LS, Kasmieh R, Wakimoto H, Hingtgen S, van de Water JA, Mohapatra G, Figueiredo JL, Martuza RL, Weissleder R, Shah K (2009). Assessment of therapeutic efficacy and fate of engineered human mesenchymal stem cells for cancer therapy. Proceedings of the National Academy of Sciences of the United States of America.

[45] Ghandhi SA, Yaghoubian B, Amundson SA (2008). Global gene expression analyses of bystander and alpha particle irradiated normal human lung fibroblasts: synchronous and differential responses. BMC medical genomics.

[46] Hei TK, Zhou H, Ivanov VN, Hong M, Lieberman HB, Brenner DJ, Amundson SA, Geard CR (2008). Mechanism of radiation-induced bystander effects: a unifying model. The Journal of pharmacy and pharmacology.

[47] Shareef MM, Cui N, Burikhanov R, Gupta S, Satishkumar S, Shajahan S, Mohiuddin M, Rangnekar VM, Ahmed MM (2007). Role of tumor necrosis factor-alpha and TRAIL in high-dose radiation-induced bystander signaling in lung adenocarcinoma. Cancer research.

[48] Luce A, Courtin A, Levalois C, Altmeyer-Morel S, Romeo PH, Chevillard S, Lebeau J (2009). Death receptor pathways mediate targeted and non-targeted effects of ionizing radiations in breast cancer cells. Carcinogenesis.

[49] Klammer H, Mladenov E, Li F, Iliakis G (2015). Bystander effects as manifestation of intercellular communication of DNA damage and of the cellular oxidative status. Cancer letters.

[50] Jing W, Chen Y, Lu L, Hu X, Shao C, Zhang Y, Zhou X, Zhou Y, Wu L, Liu R, Fan K, Jin G (2014). Human umbilical cord blood-derived mesenchymal stem cells producing IL15 eradicate established pancreatic tumor in syngeneic mice. Molecular cancer therapeutics.

[51] Aucher A, Rudnicka D, Davis DM (2013). MicroRNAs transfer from human macrophages to hepato-carcinoma cells and inhibit proliferation. Journal of immunology.

[52] Castleton A, Dey A, Beaton B, Patel B, Aucher A, Davis DM, Fielding AK (2014). Human mesenchymal stromal cells deliver systemic oncolytic measles virus to treat acute lymphoblastic leukemia in the presence of humoral immunity. Blood.

