# Supplementary material for: Human mesenchymal stem cells enhance the systemic effects of radiotherapy
Source: Oncotarget. 2015 Aug 17;6(31):31164–80. doi: 10.18632/oncotarget.5216 (PMC4741595; doi:10.18632/oncotarget.5216)
Supplement: Supplementary file 1 [file oncotarget-06-31164-s001.pdf]

Human mesenchymal stem cells enhance the systemic effects of radiotherapy

Supplementary Material

1. Mathematical model applied

The mathematical model applied for the analysis of this set of data for the growth of tumor-cell colonies (*Tcg*) as a function of time is the defined as exponential growth. From the fit of the experimental data to the equation: $Tcg = e^{kt}$ where *k* is a constant and *t* is the time we can obtain the value of the slopeand, using this, the values for the duplication time: $T_D = ln2/k$.

The Tumor cell growth *Tcg* curve in the experiments performed to study the time-course evolution of colony size under treatment with RCM 48h shows a very slow positive slope in the case of A375, whereas the slope of the growth kinetics in G361 shows a negative value indicating the progressive reduction in the surface covered by the cells colonies. Following the model proposed by [Steel, G.G. 1967] if the rate of loss of tumor cells from a tumor or colony under treatment conditions may be estimated by measuring the cell production rate in this condition and comparing this with the rate at which cells are observed to be added to the colony grown under control conditions, then it can be shown that:

$$C_L = 100 * [1 - \frac{T_{D\ control}}{T_{D\ experimental}}]$$

Where C_L is cell loss rate; and $T_{D\ (Control)}$ and $T_{D\ (Experimental)}$ are the duplication times calculated for the growth kinetics of tumor models in control and in each experimental condition.

Steel GG. Cell loss as a factor in the growth rate of human tumours. European journal of cancer 1967;3(4):381-7.

2. Dosimetric evaluation

The aim of this study has been firstly, to measure the absorbed dose on the treated tumor with extreme precision and secondly, to estimate what were the levels of the received dose in the tumors that had to be protected from the radiation action using the shield designed for evaluating the bystander effect *in vivo*.

Ionizing radiation was delivered by X-Ray TUBE (YXLON, model Y, Tu 320-D03) using a voltage of 240.4kV, a working current of 13.0 mA, a 0.32 mm Cu filter system, a 5 mm diameter focus of irradiation, a target distance of 10 cm and an irradiation field of 0.78 cm^2, and a dose rate of 1502 ± 0.3 mGy/min.

For the radiochromic film dosimetry system we have used the Gafchromic film EBT3 [1, 2] and a scanner Epson Expression 10000XL. We have applied two different assemblies, which are described in the following paragraphs.

In the first we reproduced the irradiation conditions in which is it possible to quantify the absorbed dose, taking into account the international protocols [3]. The films are irradiated in whole backscattering conditions on a water phantom at 50 cm of the X-Ray source. This allows us to verify the values of the absorbed doses using the ionometric system normally used in the calibration of X-Rays beams of medium and low energy.

The second assembly seeks to reproduce the irradiation conditions of the mice (Fig. 1). In this case the films are placed between two sheets of RW3 equivalent-water material (PTW, Friburgo, Germany), 160mm ×160mm× 10mm and 160mm × 160mm × 2mm.

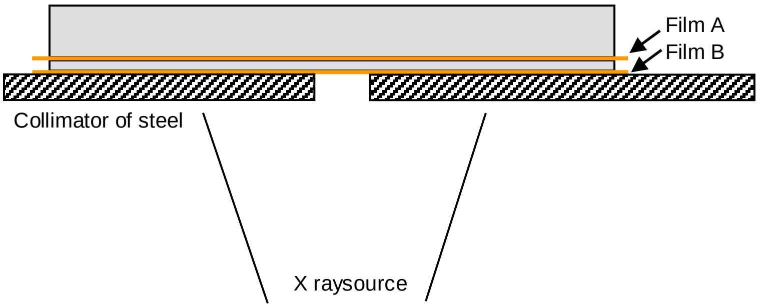

Figure 1: Assembly that mirrors the irradiation conditions of the animals (mice phantom). Film A is approximately 2.3 mm thick and the width of film B is 0.28 mm.

24 hours after the phantom irradiation we performed the scanning of the films and their analysis using ImageJ software.

Both the irradiated and the unirradiated films (control film) are scanned in the transparence mode. All the film scanning was performed in the same direction and at the same region of the scanner. We have applied the color method (48 bit), the green channel and a resolution of 96 ppp. The pixel values associated to every position are transformed to optical densities with the help of the equation:

$$\mathrm{net}OD = \mathrm{Log}_{10}\left(\frac{VP_{\mathrm{control}}}{VP_{\mathrm{exposed}}}\right) \tag{1}$$

where net OD is the net optical density VP_{control} the calculated pixel mean value for the control film after defining an area of 2x2 cm, and $VP_{exposed}$ the pixel values corresponding to each position in the irradiated films. To obtain the relative dose is it necessary to perform another mathematical transformation using the following expression [4]:

$$D = -k \frac{\mathrm{net}OD^{t}}{\ln(\mathrm{net}OD)} \qquad (2)$$

In this expression instead of the exponent of 2/3 recommended by Devic*et al.* [4], we have used our own data obtained after calibration of the film with beams of X-Rays generated by a lineal electrons accelerator with energy nominal values from 6 to 15 MV. For this type of photon the determination of absorbed doses is very exact and, considering the small dependence of the response of the film compared to the energy [1, 2] we may use the equation (1) and (2) to calculate the value of absorbed dose for the energy of 240 kVp of the photons used in this study. Therefore, we have used the values of k = 21.6 ± 0.2 and t = 0.768 ± 0.006. This uncertainty and those that appear in the next paragraphs are expressed with a coverage factor of k = 1.

Although the RW3 material for dosimetry media and low energy photon beams is not recommended, it is important to note that the differences observed between water and RW3 for energy and selected depth (2 mm) are not significant [4].

Film irradiation in reference conditions

Following the method previously described we have irradiated a film fragment at the nominal doses of 0.5, 1, 2 y 4 Gy, established by the ionometrics procedures accepted in international practice [3]. The results are summarized in Table1.

Nominal doses (Gy)	Estimated doses with the film (Gy)
0.5	0.51±0.06
1.0	0.86±0.08
2.0	1.97±0.13
4.0	3.95±0.15

Table 1.Nominal doses estimated with ionometrics methods commonly used and doses measured with the film EBT3

The mean difference between nominal doses and established doses with the film is 4%, which shows a good dosimetric concordance, taking into account the uncertainty bonded to the film.

Film irradiation in the conditions of animal treatment

We have irradiated three pairs of films placed in the positions indicated in Figure 1, we have kept another two films outside the irradiation facility that have been used as control. The results for the mean adsorbed doses measured are 2.11±0.08 Gy in the surface position (B in Fig. 1) and 2.00±0.08 Gy for the film placed at 2.3 cm of depth (A in Fig. 1).

Figure 2 shows a representative example of the dosimetric profile obtained for a film placed at 2 mm depth.

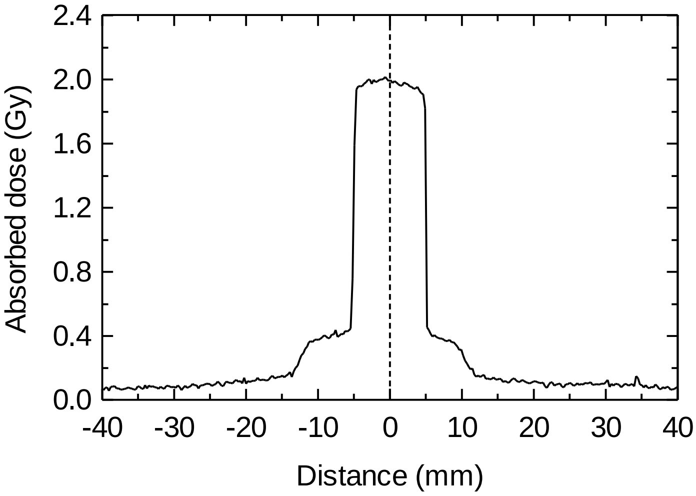

Figure 2.Dose distribution measured with a strip of film irradiated at 2.3 cm depth according to the assembly plotted in Figure 1. The distance origin has been taken at the center of the hole made in the steel sheet used as shielding.

To sum up we can state that the absorbed dose in the tumor is approximately, 2 Gy, whereas the dose received in the contra-lateral tumor is approximately 0.1 Gy.

1. Brown, T.A., et al., *Dose-response curve of EBT, EBT2, and EBT3 radiochromic films to synchrotron-produced monochromatic x-ray beams.* Med Phys, 2012. **39**(12): p. 7412-7.
2. Sorriaux, J., et al., *Evaluation of Gafchromic(R) EBT3 films characteristics in therapy photon, electron and proton beams.* Phys Med, 2013. **29**(6): p. 599-606.
3. Hill, R., et al., *Advances in kilovoltage x-ray beam dosimetry.* Phys Med Biol, 2014. **59**(6): p. R183-231.
4. Devic, S., et al., *Linearization of dose-response curve of the radiochromic film dosimetry system.*Med Phys, 2012. **39**(8): p. 4850-7.

3. Direct and bystander radiation effects. Mathematical and biophysical model

From a theoretical point of view the adjuvant role of MSC to radiotherapy might be thought of as a potential tool to increase the weight of the second factor of our mathematical model for biological radiotherapy action. (Gómez-Millan et al. 2012; Lara et al. 2015)

$$S = e^{-(\alpha D+\beta D^2)} \cdot \left\{1 - \chi_{max} \cdot \frac{D}{K_{By} + D}\right\}$$

where S is surviving fraction, D dose, α and β the coefficients of lineal-quadratic model, χ represents cell death produced by bystander effect, χ_{max} is maximum cell death, d the dose at which the bystander effect was measured and K_{By} the dose administered to get a bystander effect for which the cell-death rate is half maximum.

Thus, we have proposed that when radiation therapy is applied to a tumor-cell population the cells can be classified into four divisions:

A) Dead cells: cells that have undergone irreparable lesions, (this is the lethal-lesion compartment in Curtis's model);

B) Committed cells: cells affected by potentially lethal lesions, the eventual fate of which depends on competing processes of repair and mis-repair, (these are the potentially lethal lesions in the lineal-quadratic equation).

C) Activated cells: cells that are either minimally damaged or have been able to repair their lesions to a level of residual damage compatible with survival. These cells might become an active source of cytokines, reactive oxygen species (ROS) and reactive nitrogen species, thus affecting the total burden of tumor cells via diffusion and systemic circulation, (this is the bystander component of radiation induced cell death);

D) Undamaged cells: surviving cells after each fraction of dose, which need to be controlled with successive irradiation treatments. Clearly, the tumor control probability is dependent, moreover on the size of this compartment, on the dose per fraction, total dose, time, toxic conditions and cellular radio-sensitivity.

The cell population (C) is involved in short-range bystander effects by means of direct gap-junction including intra-and inter-cellular communication involving free radicals, and also in long-range bystander effects, which occur at a distance from the irradiated tumor. The long-range bystander effect produced by cytokines released into the lymphatic or vascular circulation might materially modify conventional expectation in radiotherapy by producing systemic or abscopal positive effects.

And this is biologically conceivable, because with the MSCs activated by radiation it is possible to increase the amount of cytotoxic proteins, amongst them TRAIL and DKK3, released by the MSCs *in situ*, moreover, the radiation applied on the tumor increased the expression of the *TRAIL* death receptor *DR5*. The up-regulation of these proteins may

produce the increase of tumor-cell death and the inhibition of cell proliferation via the potentation of the bystander effect and the global results are the synergistic action observed between RT and MSC.

Gomez-Millan J, Katz IS, Farias Vde A, Linares-Fernandez JL, Lopez-Penalver J, Ortiz-Ferron G, et al. The importance of bystander effects in radiation therapy in melanoma skin-cancer cells and umbilical-cord stromal stem cells. Radiotherapy and oncology : journal of the European Society for Therapeutic Radiology and Oncology 2012;102(3):450-8.

Lara PC, Lopez-Penalver JJ, Farias VD, Ruiz-Ruiz MC, Oliver FJ, Ruiz de Almodovar JM. Direct and bystander radiation effects: A biophysical model and clinical perspectives. Cancer letters 2014;356:5-16.

4. Tumor cell lines.

The A375 cell line was a gift from Dr.Bosserhoff (Institute of Pathology, Regensburg University, Germany). G361 cells were obtained from the cell-culture facility at the University of Granada (CIC) (ref # ECCAC: 8803040).

5. Quantitative Real-Time PCR

This method allowed us to access the optical density values corresponding to TRAIL and DKK3 protein content in the cells. The human-specific primers used for relative quantitation of the gene expression of *TRAIL,* TRAIL receptor *DR5* and *DKK3* mRNA expression in each cell line by quantitative Real-Time PCR were:

TRAIL (w:TAGCAGCTCACATAACTGGGAC; rev: AGCCTTTTCATTCTTGGAGTTTGG), TRAIL receptor *DR5* (fw: CCAGCAAATGAAGGTGATCCCA; Rev: CATTGTCCATGAGGCCCAACT) and

DKK3 (fw: TCATCACCTGGGAGCTAGAG;

rev: CAACTTCATACTCATCGGGG)

We used *RPLP0* (fw: CAGATTGGCTACCCAACTGTT;

rev: GGCCAGGACTCGTTTGTACC) mRNA levels as internal reference gene to normalize the data.

6. ELISA of umbilical cord MSC whole-cell assay

Images of the wells were treated with the "Threshold colour" tool to select only colonies larger than 50 cells and then transformed to binary to allow particle analysis. The colony number and size was determined for each experiment measuring all colonies which were bigger than 50 cells. To take this into consideration we used the "Total Area" parameter returned by the ImageJ's Particle Analysis tool and the total area occupied by the colonies in each experimental condition was compared with the colonies from the untreated condition. Briefly, the cells in exponential growth were harvested, counted, and seeded in an appropriate number on 24-well plates. Cells were left for 24 hours to adhere to the culture flasks and then fixed in 4% PFA for 10 minutes at room-temperature, washed with PBS three times and stored at 4 ºC until used. The cells were then permeabilized by incubating them with PBS with 0.1 % saponin at room temperature for 10 minutes, washed three times with PBS with 0,05% Tween-20 (PBSt) and then incubated with PBS with 1% BSA for 1 hour at room temperature to block unspecific epitopes. After washing thoroughly with PBSt, cells were incubated with a biotinilated antibody against TRAIL (BD, 550948c) or against DKK3 (R&D, 842226) mixed with the steptavidin conjugated to horseradish-peroxidase for 1 hour at room temperature. Cells were then washed at least 7 times with PBSt, incubated at room temperature, protected from light for 30 minutes with the freshly prepared substrate solution (BD OptEIA™, 555214). The reaction was stopped with phosphoric acid 1 M.

Samples were monitored at 450 nm with an automatic plate reader (TitertekMultiskan plus, ICN Flow).

7. Tumor volume calculation

Tumor volume was calculated using the formula $v = \left(\frac{\pi}{6}\right)(a^2 \cdot b)$, a and b being the values for the smaller and the larger diameter, respectively. Mice bearing tumors larger than 64 mm^3 were randomly distributed into 4 different groups: control, radiotherapy, MSC therapy and radiotherapy plus MSC therapy.

7. Histopathological and immunohistochemical studies

Tumors from a human melanoma cell line were xenografted in 32 mice (8 from each study group) and were immediately fixed in 10% buffered formalin for 48 h, and then embedded in paraffin, and 4 µm sections were dewaxed, hydrated, and stained using the hematoxylin-eosin technique. On these slides we determined the mitotic index, the necrotic areas and apoptotic cells observed outside the necrotic fields. Moreover, a complete microscopic study of pelvic, abdominal and thoracic organs was done to assess possible metastasis. For further details see supplementary materials.

The mitotic index was determined by counting the number of mitotic figures under microscopic observation in 10 high power fields (40x objective) from each of the 8 mice from each group, following the procedure previously described [Evans A.T. 1992]. Data were represented as mean±SEM for each of the groups studied (Fig. 7). Besides the mitotic index determination, data of the tumors from the whole section were gathered on the semi-quantitative score (0: absent, 1: low, 2: medium, and 3: intense) to identify the necrosis zones observed, and the number of apoptotic cells placed outside the necrotic fields. We assessed 10 randomly selected microscopic fields obtained from

64 different slides with 3 serial sections in each from all mice with tumors and compared the tumor size and treatment. Finally, a complete microscopic study of the pelvic, abdominal and thoracic organs was done to assess possible metastasis.

Paraffin-embedded sections were dewaxed, hydrated, and heat-treated in 1 mM EDTA pH 8 for antigenic unmasking in an antigen retrieval PT module (Thermo Fisher Scientific Inc., Waltham, MA) at 95°C for 20 min. Sections were incubated for 10 min at room temperature with pre-diluted monoclonal antibodies against Mart-1/Melan A (clones M2-7C10+M2-9E3) and Ki-67 (clone SP-6), and polyclonal antibody against CD90 (Thy-1). All the antibodies and reagents were purchased from Master Diagnóstica, Granada, Spain. An appropriate isotype for each antibody was used as a negative control. The immunohistochemical study was conducted in an automatic immunostainer (Autostainer 480, LabVision Fremont, CA) using the micropolymer-peroxidase-based method (UltravisionQuanto, Master Diagnóstica, Granada, Spain), followed by development with diaminobenzidine. A semiquantitative study of the different immunostainings was done. Results were expressed in percentages of positivity.

Evans AT, Blessing K, Orrel JM, Grant A. Mitotic Indices, Anti-PCNA immunostaining and AgNORs in thick cutaneous melano-mas displaying paradoxical behavior. J Pathol 1992; 168: 15-22
